# PSMC5, a 19S Proteasomal ATPase, Regulates Cocaine Action in the Nucleus Accumbens

**DOI:** 10.1371/journal.pone.0126710

**Published:** 2015-05-11

**Authors:** Yoko H. Ohnishi, Yoshinori N. Ohnishi, Takanori Nakamura, Mizuki Ohno, Pamela J. Kennedy, Ohkawa Yasuyuki, Akinori Nishi, Rachael Neve, Teruhisa Tsuzuki, Eric J. Nestler

**Affiliations:** 1 Fishberg Department of Neuroscience, Icahn School of Medicine at Mount Sinai, New York, New York, United States of America; 2 Department of Pharmacology, Kurume University School of Medicine, Kurume, Fukuoka, Japan; 3 The Research Support Center, Research Center for Human Disease Modeling, Graduate School of Medical Sciences, Kyushu University, Fukuoka, Japan; 4 Department of Medical Biophysics and Radiation Biology, Faculty of Medical Sciences, Kyushu University, Fukuoka, Japan; 5 Department of Advanced Medical Initiatives, Division of Epigenetics, Faculty of Medicine, Kyushu University, Fukuoka, Japan; 6 Department of Brain and Cognitive Sciences, Massachusetts Institute of Technology, Cambridge, Massachusetts, United States of America; University of Leicester, UNITED KINGDOM

## Abstract

ΔFosB is a stable transcription factor which accumulates in the nucleus accumbens (NAc), a key part of the brain’s reward circuitry, in response to chronic exposure to cocaine or other drugs of abuse. While ΔFosB is known to heterodimerize with a Jun family member to form an active transcription factor complex, there has not to date been an open-ended exploration of other possible binding partners for ΔFosB in the brain. Here, by use of yeast two-hybrid assays, we identify PSMC5—also known as SUG1, an ATPase-containing subunit of the 19S proteasomal complex—as a novel interacting protein with ΔFosB. We verify such interactions between endogenous ΔFosB and PSMC5 in the NAc and demonstrate that both proteins also form complexes with other chromatin regulatory proteins associated with gene activation. We go on to show that chronic cocaine increases nuclear, but not cytoplasmic, levels of PSMC5 in the NAc and that overexpression of PSMC5 in this brain region promotes the locomotor responses to cocaine. Together, these findings describe a novel mechanism that contributes to the actions of ΔFosB and, for the first time, implicates PSMC5 in cocaine-induced molecular and behavioral plasticity.

## Introduction

ΔFosB, a truncated product of the *FosB* gene, belongs to the Fos family of transcription factors, which also include c-Fos, full-length FosB, Fra-1, and Fra-2. ΔFosB, like other Fos proteins, heterodimerizes with a Jun family protein—c-Jun, JunB, or JunD—to form an active AP-1 (activator protein-1) transcription factor complex, which induces or represses the expression of specific target genes [1,2].

ΔFosB has been shown to play a key role in drug addiction [2]. Uniquely among Fos family proteins, it accumulates in nucleus accumbens (NAc) and other reward-related brain regions after repeated drug administration due to its high level of stability [3,4], which is mediated by the lack of C-terminal degron domains and by phosphorylation by several proteins kinases [5–7]. Such induction of ΔFosB in the NAc mediates increased behavioral responses to drugs of abuse. Thus, overexpression of ΔFosB in this brain region of adult animals, either by use of viral vectors or inducible bitransgenic mice, increases an animal’s sensitivity to the locomotor-activating and rewarding effects of cocaine and opiates as well as an animal’s motivation to self-administer cocaine [7–11]. Conversely, overexpression of dominant negative antagonists of ΔFosB causes the opposite behavioral phenotypes [10–12].

We and others have previously confirmed, by use of gel shift assays, that JunD and perhaps other Jun family proteins are the major binding partners of ΔFosB in the brain in vivo [13–15]. However, to date, there has not been an open-ended, unbiased evaluation of ΔFosB’s binding partners in brain. Here, we sought to identify novel binding partners for ΔFosB by use of a yeast two-hybrid assay [16,17]. Our data revealed that PSMC5, also known as SUG1, is a robust partner of ΔFosB both in vitro and in the NAc in vivo, where it joins ΔFosB as part of a cocaine-induced transcription activation complex, which also contains CBP (CREB binding protein) and p300—both histone acetyltransferases (HATs)—as well as BRG1 (a chromatin remodeling protein). We go on to show that chronic exposure to cocaine alters the nuclear levels of PSMC5, an ATPase-containing subunit of the 19S proteasomal complex, in the NAc and that PSMC5 in turn controls behavioral responses to cocaine.

## Material and Methods

### Yeast two-hybrid screening

MaV203 yeast cells (Invitrogen Life Technologies) were co-transfected with pDBLeu driving different fragments of the ΔFosB protein, and a mouse brain library was subcloned in pPC86 (Invitrogen Life Technologies). Transformed cells were grown on SC-medium lacking leucine, tryptophan, and histidine, and containing 10 mM of 3-aminotriazole. Binding between FosB fragments and a candidate partner induces three reporter genes (*His3*, *Ura3*, and *LacZ*), and the induction makes transformants able to survive under the cultured conditions used. Positive clones were retested with fresh pDBLeu-ΔFosB fragments by retransformation assays in MaV203 cells.

### Cell lines

Mouse Neuro 2A neuroblastoma cells (ATCC) were maintained in Eagle's minimum essential medium (EMEM) (ATCC), supplemented with 10% fetal bovine serum (FBS) at 37°C and 5% CO_2_. Rat 1A cells were a gift from Yusaku Nakabeppu (Fukuoka, Japan) [18] and maintained in Dulbecco’s MEM (DMEM) (Life Technologies), supplemented with 10% FBS at 37°C and 5% CO_2_. Transfection of cells with plasmid DNA was accomplished using Effectene (Qiagen) according to the manufacturer’s instructions.

### PSMC5 and ΔFosB constructs

**Fig 1:**
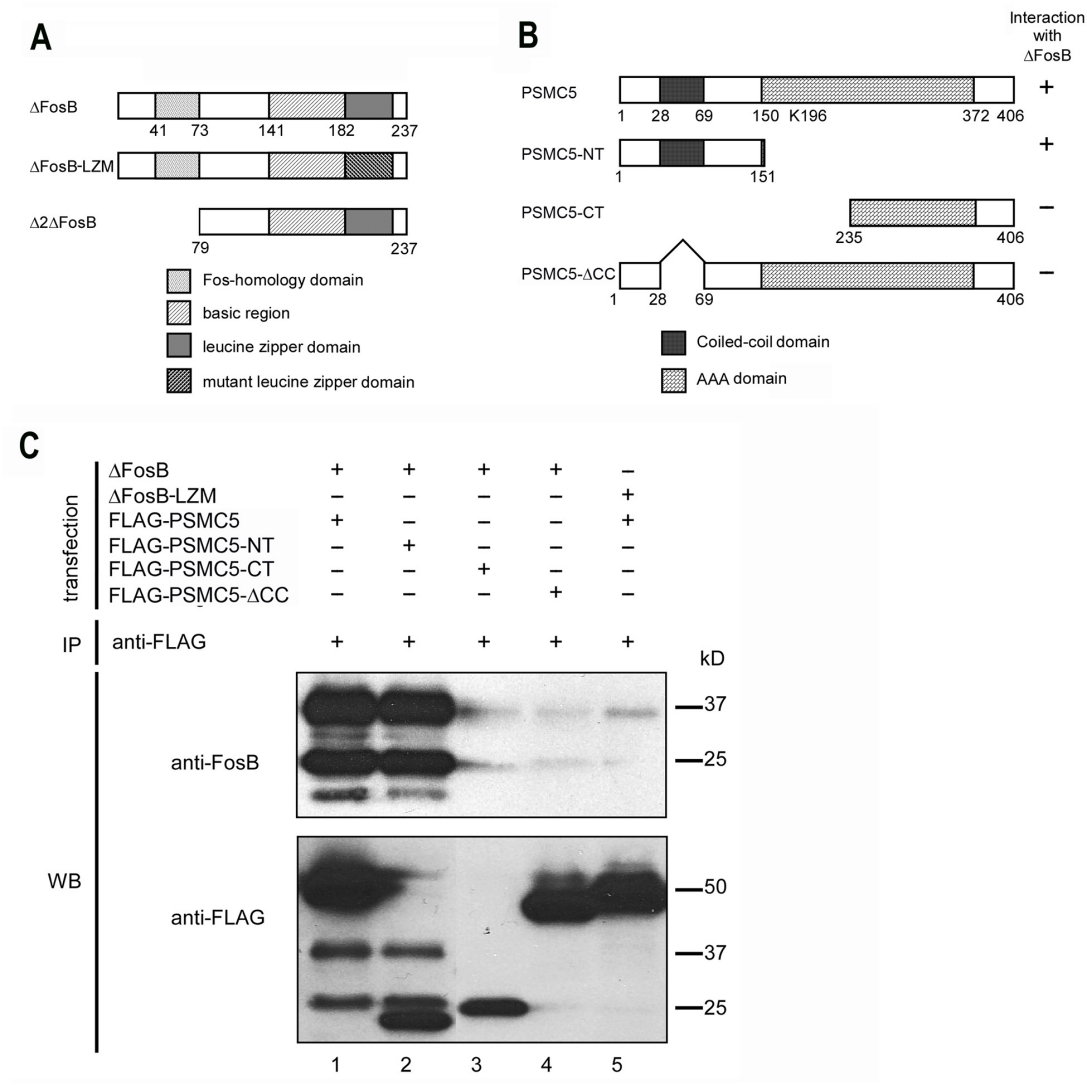
ΔFosB binds to PSMC5 in vitro. A. Schematic of ΔFosB, ΔFosB-LZM in which the leucine zipper domain is mutated to obliterate ΔFosB heterodimerization with Jun proteins, and Δ2ΔFosB which lacks the first 78 amino acids of the ΔFosB N-terminus. B. Schematic of PSMC5, PSMC5-NT which comprises the first 151 amino acids of PSMC5, PSMC5-CT which lacks the first 235 amino acids of PSMC5, and PSMC5-ΔCC which lacks the coiled-coil domain (amino acids 28–68). The AAA domain corresponds to a motif, ATPases Associated with diverse cellular Activities, present in many ATPases. C. 2.4 μg of pcDNA3.1-ΔFosB (lanes 1–4) or ΔFosB-LZM (lane 5) was co-transfected with 2.4 μg of FLAG-tagged PSMC5 or various deletion mutants into Neuro2a cells. Two days after transfection, cells were lysed and subjected to immunoprecipitation with an anti-FLAG antibody and then Western blotted with anti-ΔFosB or anti-FLAG antibody. Note that ΔFosB, but not ΔFosB-LZM, binds robustly to PSMC5 or PSMC5-NT, but not PSMC5-CT or PSMC5-ΔCC. The data shown in the figure were replicated in triplicate in each of three separate experiments.

Several mutant forms of PSMC5, each FLAG-tagged at their N-terminus, were generated for use in immunoprecipitation or viral-mediated gene transfer experiments. These included: PSMC5-K196M, PSMC5-Δcoiled-coil domain (PSMC5-ΔCC, lacking amino acids 27–68), PSMC5-NT (consisting of the N-terminal fragment of the protein, amino acids 1–151), and PSMC5-CT (consisting of the C-terminal fragment of the protein, 172 amino acids) (see Fig 1). We also utilized N-terminal MYC-tagged forms of wildtype ΔFosB as well as ΔFosB with a mutation in its leucine-zipper domain (mutation of amino acids 182 to 205 which is known to obliterate heterodimerization with Jun family proteins [6].

### Animals

Nine- to 11-week-old C57BL/6J male mice (The Jackson Laboratory) were used for all experiments. Animals were housed on a 12-h light-dark cycle with access to food and water *ad libitum* and were habituated 1 week before experimentation. Two cocaine treatment regimens were used. To study the biochemical effects of cocaine, animals were given 7 daily doses of cocaine (20 mg/kg) or saline, and killed by decapitation 24 hr after the last injection. This is a standard protocol, which has been shown to produce numerous molecular and cellular responses to the drug [7]. To study the influence of PSMC5 in nucleus accumbens on behavioral responses to cocaine, we used a subthreshold dose of the drug (7.5 mg/kg; see Locomotor sensitization below) based on the hypothesis that PSMC5 would, like ΔFosB, increase an animal’s sensitivity to cocaine [8]. All animal experiments were approved by the Institutional Animal Care and Use Committee at Mount Sinai.

### Immunoprecipitation and Western blotting

Neuro 2A cells were transfected with wildtype or mutant forms of PSMC5. Two days after transfection, cells were washed in PBS, lysed in RIPA buffer (50 mM Tris pH 7.4, 150 mM NaCl, 1 mM EDTA, 1% NP-40, 0.25% sodium deoxycholate, 10 mM sodium butyrate, protease inhibitor cocktail). Lysates were split and incubated with either nonimmune IgG (Sigma) or anti-FLAG antibodies (Sigma) for 3 hr at 4°C. Immunoprecipitation was performed with Protein G beads (Invitrogen) as described [19]. Briefly, immunoprecipitated proteins were subjected to SDS-PAGE and analyzed by Western blotting using anti-FosB/ΔFosB antibody (Cell Signaling Technology) based on published protocols [7]. For in vivo protein binding assays, we used purified nuclear fractions from punch-dissected NAc of mice after chronic cocaine treatment (20 mg/kg IP daily for 7 days, with mice used 24 hr after the last injection). Co-immunoprecipitation from nuclear fractions was performed using the Nuclear Complex Co-IP kit (Active Motif) following the manufacturer’s instructions. The following antibodies were used: MYC or ß-actin, Cell Signaling Technology (Danvers, MA), PSMC5 and histone H3, Abcam (Cambridge, MA), CBP, p300 and BRG1, Santa Cruz Biotechnology (Santa Cruz, CA), and FLAG M2, Sigma.

### Immunohistochemistry

Immunohistochemistry was performed according to published procedures [20]. Mice were anesthetized and perfused intracardially with 4% paraformaldehyde in PBS. Brain were cryoprotected with 30% sucrose, and then frozen and stored at -80°C until use. Coronal sections (40 μm) were cut on a cryostat and processed for immunohistochemistry. Free-floating sections were pre-incubated in a blocking buffer containing 0.3% Triton and 3% normal donkey serum. ΔFosB was detected using a goat polyclonal antibody raised against the N-terminal portion of the protein (1/1000 Santa Cruz Biotechnology). PSMC5 was detected using a rabbit polyclonal antibody (1/100 Abcam, Cambridge, MA). Images were taken with a confocal microscope (60x magnification; Zeiss).

### Locomotor sensitization

All mice received daily IP injections of saline for 3 days to habituate them to the stress of the injections. The next day, mice were injected IP with saline or a subthreshold dose of cocaine (7.5 mg/kg; see under Animals above) and placed immediately into novel locomotor boxes. The locomotor activity of the mice was recorded using a photobeam system as ambulatory beam breaks for 30 min. These treatments were repeated daily for 3 days.

### Viral-mediated gene transfer

We used extensively published methods for viral-mediated gene transfer [7,8,11,19]. Briefly, expression plasmids for PSMC5 or for several of its mutants (see PSMC5 and ΔFosB constructs above) were subcloned into the bicistronic p1005(+) HSV plasmid expressing GFP under the control of the CMV promoter, and PSMC5 or its mutants under that of the IE4/5 promoter. Under ketamine (100 mg/kg)/xylazine (10 mg/kg) anesthesia, mice were positioned in a small-animal stereotaxic instrument, and the cranial surface was exposed. Thirty-three gauge syringe needles were used to bilaterally infuse 0.5 μl of an HSV vector into the NAc at a 10° angle (AP +1.6; ML +1.5; DV -4.4) at a rate of 0.1 μl/min. Animals receiving HSV injections were allowed to recover for 2 days following surgery before experimentation.

### Statistics

ANOVAs and student’s t-tests were used, corrected for multiple comparisons, with significance set at p<0.05.

## Results

### PSMC5: novel binding partner of ΔFosB

We performed preliminary experiments to identify an appropriate fragment of ΔFosB that served as bait in a yeast two-hybrid assay without autoactivating the system. Holo-ΔFosB induced reporter gene activity on its own, as did the N-terminal 1–78 amino acid fragment of the protein. However, an N-terminal truncated ΔFosB (Fig 1A), termed Δ2ΔFosB, which lacks the first 78 amino acids of the protein, did not have this effect. Therefore, we used Δ2ΔFosB as the bait protein.

To screen for potential binding partners, we used a mouse brain library subcloned in pPC86. We identified 11 candidates for binding partners. Although these proteins included ΔFosB’s known heterodimerization partners, c-Jun and JunD (Table 1), the most prevalent candidate by far was PSMC5. While surprising, this was an interesting finding, since PSMC5 was shown in a single report years ago to bind to c-Fos in vitro [21]. However, there are no prior reports of PSMC5 involvement in cocaine action. Nevertheless, because of the strength of the PSMC5 signal in the yeast two-hybrid assay, we set out to further study possible ΔFosB-PSMC5 interactions.

**Table 1:** Results of Yeast Two-Hybrid Screening with Δ2ΔFosB.

Gene symbol	Protein name	Number of clones	x-Gal	sc-URA	sc-HIS
*Psmc5*	Proteasome 26S subunit, ATPase 5*	24	White	+	+++
*Atf4*	Activating transcription factor 4	6	Blue	++	++
*Optn*	Optineurin (Huntingtin-interacting protein L)	3	White	++	+++
*Pscd2*	Pleckstrin homology, Sec7 and coiled-coil domains 2	1	White	+	+++
*Yeats4*	YEATS domain containing 4	1	Blue	+	+++
*Nin*	Ninein (GSK3ß interacting protein	1	Blue	+	+++
*Cep250*	Centrosomal protein 250 kD	1	White	+	+++
*Ankrd35*	Ankyrin repeat domain 35	1	White	+	+
*Lrrfip2*	Leucine rich repeat (in FLII) interacting protein 2	1	White	+	+
*Jun*	c-Jun transcription factor	1	Blue	+++	+
*Jund*	JunD transcription factor	1	White	++	+++

*Although PSMC5 is categorized as a subunit of the 26S proteasome, the 26S proteasome is composed of 19S and 20S particles. PSMC5 is a subunit of the 19S particle, which contains the ATPase activity of the proteasome.

First, to confirm the physical interaction between ΔFosB and PSMC5, we performed in vitro co-immunoprecipitation experiments. We found that FLAG-tagged PSMC5 (Fig 1B), transfected into Neuro 2A cells, effectively pulled down ΔFosB (Fig 1C). Second, to identify the region in PSMC5 that is responsible for its binding to ΔFosB, we generated several FLAG-tagged PSMC5 mutants (Fig 1B) and repeated the co-immunoprecipitation experiment. ΔFosB was pulled down effectively with the N-terminal 151 amino acids of PSMC5 (PSMC5-NT), but not with the C-terminal 172 amino acid fragment of the protein (PSMC5-CT) (Fig 1C). PSMC5 lacking its coiled coil domain (PSMC5-ΔCC) was also ineffective in precipitating ΔFosB. These findings suggest that PSMC5 binds ΔFosB via its coiled-coil domain (amino acids 27–68). Moreover, FLAG-tagged PSMC5 did not precipitate a mutant form of ΔFosB with a mutated leucine zipper domain (ΔFosB-LZM) (Fig 1C), indicating that ΔFosB either binds PSMC5 through this domain or, more likely, that ΔFosB heterodimerization is required for PSMC5 binding.

### PSMC5-ΔFosB binding in NAc after chronic cocaine administration

**Fig 2:**
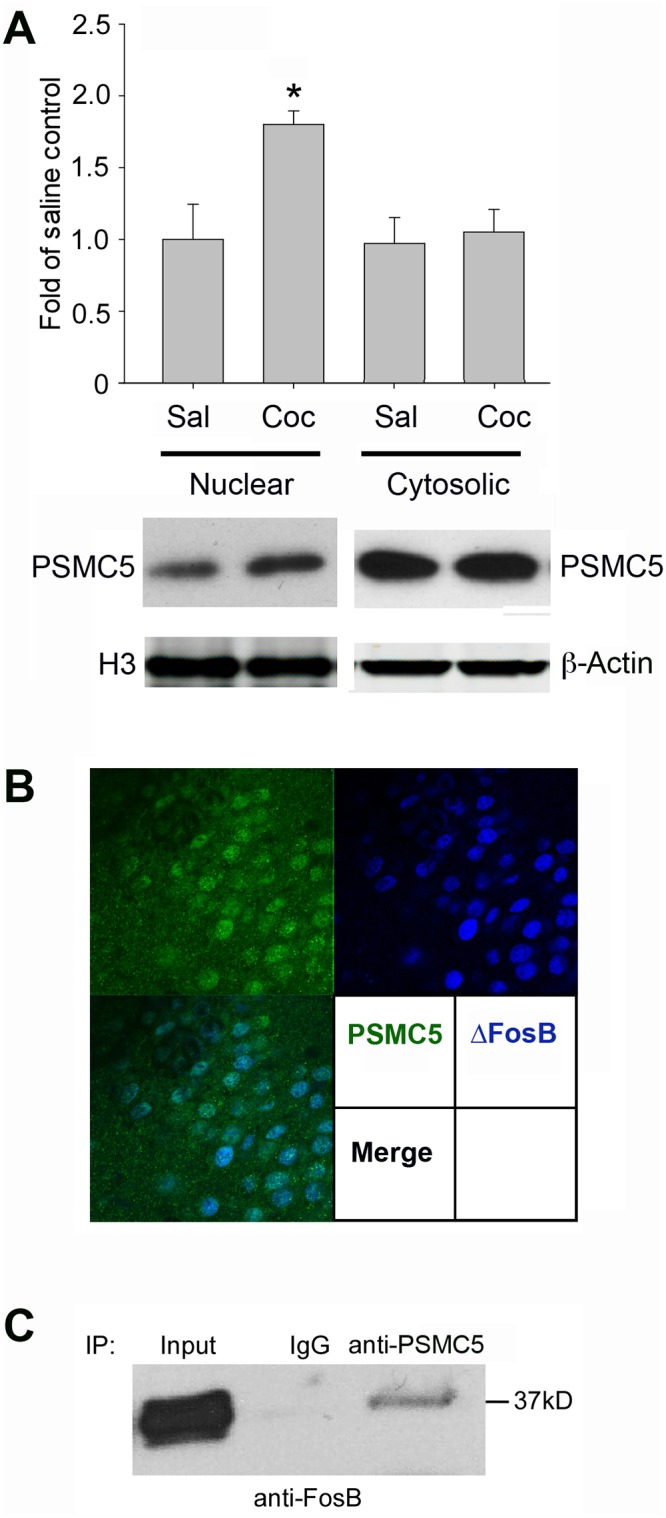
PSMC5 regulation in mouse NAc. A. Western blotting of nuclear and cytosolic fractions of NAc of mice treated daily with saline or cocaine (20 mg/kg) for 7 days, with animals analyzed 24 hr after the last injection. Cocaine increases nuclear but not cytosolic levels of PSMC5. Histone H3 and ß-actin, which were not affected by cocaine, were used as loading controls. Data are mean ± SEM (n = 8–10/group, *p<0.05). B. Co-localization of endogenous PSMC5 (green) and ΔFosB (blue) in NAc of mice treated chronically with cocaine as in A. C. Nuclear lysates of mouse NAc after chronic cocaine treatment were subjected to immunoprecipitation with anti-PSMC5 antibody or mouse IgG as control, and then Western blotted with anti-FosB/ΔFosB antibody. The figure demonstrates PSMC5-ΔFosB interactions in the NAc in vivo. Data in B and C were replicated in triplicate in each of three separate experiments.

Based on these findings in vitro, we studied whether PSMC5 levels in the NAc are altered in response to chronic cocaine administration. We found by subcellular fractionation and Western blotting that chronic cocaine increases nuclear levels of PSMC5 in this brain region without a change in cytoplasmic levels (Fig 2A). This effect was not seen after single doses of cocaine (data not shown). We next examined the localization of PSMC5 and ΔFosB in NAc by confocal immunofluorescence microscopy. We analyzed mice 24 hr after the last repeated dose of cocaine, a time point when ΔFosB is the only detectable *FosB* gene product (see Nestler 2008). We found strong PSMC5 immunoreactivity in the NAc, including a strong nuclear signal. ~85% of ΔFosB+ nuclei co-stained for PSMC5 (Fig 2B). Additionally, we performed co-immunoprecipitation experiments on NAc extracts and found that, after chronic cocaine treatment, ΔFosB was pulled down effectively by an anti-PSMC5 antibody (Fig 2C). In contrast, analysis of drug-naïve NAc (after repeated saline injections) revealed no detectable ΔFosB pull down (data not shown). These data are consistent with our findings in cell culture and confirm that ΔFosB and PSMC5 interact in the NAc in vivo.

### PSMC5 enhances ΔFosB expression in vitro

**Fig 3:**
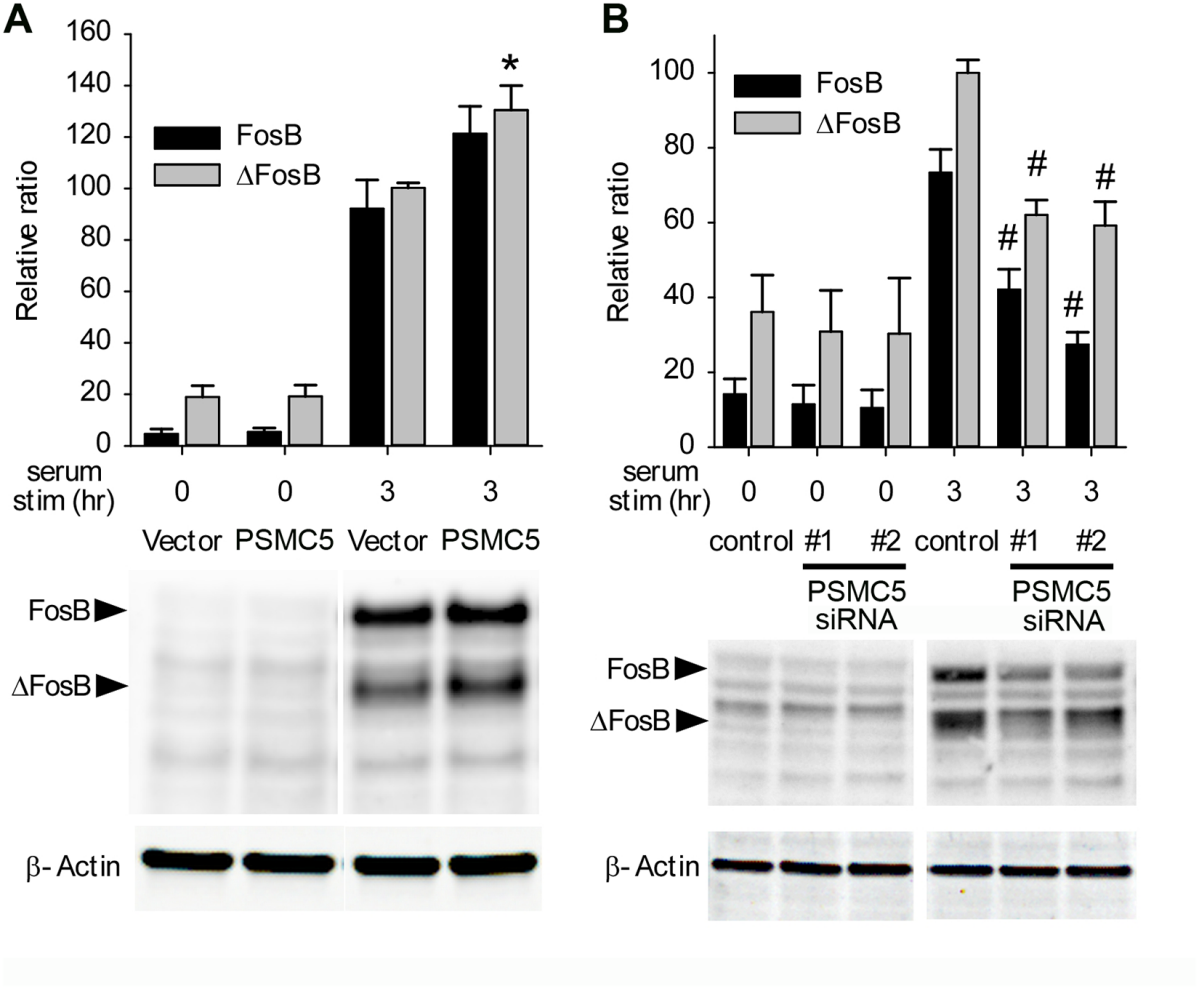
PSMC5 regulation of FosB/ΔFosB expression in Rat 1A cells. A. Rat 1A cells were transfected with 4 μg of PSMC5 or control DNA. PSMC5 overexpression had no effect on basal expression levels of FosB or ΔFosB protein as determined by Western blotting, but produced a small but significant increase in the induction of ΔFosB by serum stimulation (F(2,21) = 9.75, p = 0.001). B. Rat 1A cells were transfected with 5 pmol of either of two siRNAs or scrambled RNA (control). Both siRNAs effectively decreased PSMC5 protein levels compared to control conditions (siRNA #1, 23 ± 5% of control; siRNA #2, 18 ± 6%; p<0.05; n = 4). PSMC5 knockdown had no effect on basal levels of FosB or ΔFosB but attenulated the induction of both FosB and ΔFosB by serum stimulation (FosB: F(2,6) = 20.99, p = 0.002; ΔFosB: F(2,6) = 22.83, p = 0.002).

Since PSMC5 is a known member of the proteasome complex, we tested whether it regulates ΔFosB levels using Rat 1A cells. PSMC5 overexpression had no effect on basal levels of ΔFosB, but caused a small but significant enhancement of ΔFosB induction upon serum stimulation of the cells (Fig 3A). A similar trend was seen for full-length FosB but the effect did not achieve statistical significance. Conversely, suppression of endogenous PSMC5 expression in Rat 1A cells, achieved by use of siRNAs that target PSMC5, did not affect basal ΔFosB levels but strongly inhibited ΔFosB induction by serum stimulation (Fig 3B). Similar effects were seen for full-length FosB. These data suggest that PSMC5 does not promote the proteasomal degradation of ΔFosB, as might be expected as a core subunit of the proteasome, but instead is required for maximal accumulation of *FosB* gene products in vitro, perhaps through stabilizing the proteins.

### ΔFosB and PSMC5 form complexes with CBP, p300, and BRG1 in NAc

To better understand the transcriptional mechanisms by which PSMC5 might influence ΔFosB function, we investigated possible additional binding partners for the two proteins in the NAc under chronic cocaine-treated conditions. There is one report that PSMC5 binds to CBP—a HAT—and increases histone H3 acetylation at the MHC-II proximal promoter in HeLa cells [22]. Moreover, mice that are deficient in CBP display reduced behavioral sensitivity to cocaine as well as reduced histone acetylation at the *FosB* promoter [23]. We thus tested whether PSMC5 might bind with ΔFosB as part of complexes that also contain CBP and perhaps other transcriptional activators.

**Fig 4:**
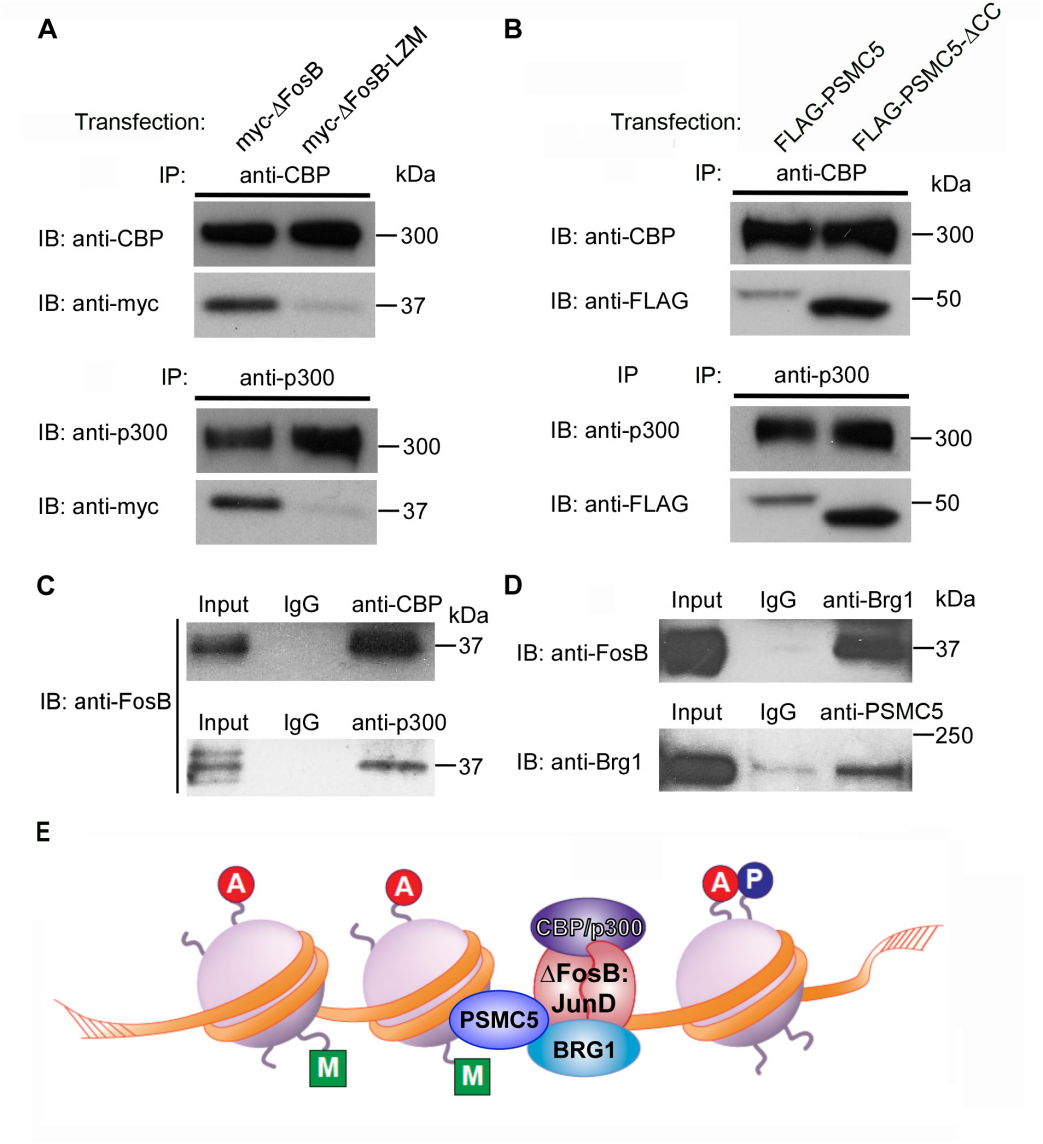
ΔFosB and PSMC5 interact with CBP, p300, and BRG1 in vitro and in vivo. A. Neuro2A cells were transfected with 2.4 μg of MYC-tagged ΔFosB or MYC-tagged ΔFosB-LZM. Cell extracts were immunoprecipitated with anti-CBP or anti-p300 antibody, and precipitates were Western blotted with the same antibody or with anti-MYC antibody. Both CBP and p300 interact with ΔFosB and such interactions require an intact leucine zipper. B. Neuro2A cells were transfected with 2.4 μg of FLAG-tagged PSMC5 or FLAG-tagged PSMC5-ΔCC. Cell extracts were immunoprecipitated with anti-CBP or anti-p300 antibody, and precipitates were Western blotted with the same antibody or with anti-FLAG antibody. Both CBP and p300 interact with PSMC5 and such interactions do not require the CC domain. C. Nuclear lysates of mouse NAc after chronic cocaine treatment were subjected to immunoprecipitation with anti-CBP or anti-p300 antibody. Subsequent Western blotting of resulting precipitates with anti-FosB/ΔFosB antibody showed endogenous interactions between ΔFosB and CBP/p300. D. Aliquots of the same nuclear lysates were subjected to immunoprecipitation with anti-BRG1 or anti-PSMC5 antibody, followed by Western blotting of precipitates with anti-FosB/ΔFosB or anti-BRG1 antibody. The results show endogenous interactions between ΔFosB and BRG1, and BRG1 and PSMC5. E. Schematic illustration of transcriptional activation complex composed of ΔFosB:JunD heterodimers interacting with CBP/p300, BRG1, and PSMC5.

We first demonstrated that ΔFosB effectively pulled down both CBP and p300, a related HAT, in Neuro2A cells (Fig 4A). In contrast, the leucine zipper mutant form of ΔFosB, as expected, did not exhibit this activity. Similarly, PSMC5 effectively pulled down CBP and p300 (Fig 4B). Interestingly, this effect was also seen for PSMC5-ΔCC, which did not pull down ΔFosB, indicating that PSMC5 interacts with CBP and p300 via other domains of the protein and independently of its binding to ΔFosB.

To confirm that these interactions also occur in vivo, we administrated cocaine chronically to induce ΔFosB and nuclear PSMC5 levels, then immunoprecipitated NAc extracts with anti-CBP or anti-p300 antibodies. Consistent with our cell culture data, immunoprecipitation of CBP or of p300 effectively pulled down ΔFosB (Fig 4C). We tested whether BRG1, a core subunit of the SWI-SNF chromatin remodeling complex, might also bind to ΔFosB and PSMC5, based on our earlier finding that BRG1 is recruited to certain ΔFosB target genes in concert with their activation in NAc after chronic cocaine [24]. We found that immunoprecipitation of BRG1 pulled down ΔFosB in NAc extracts, and that immunoprecipitation of PSMC5 likewise coprecipitated endogenous BRG1 (Fig 4D). Taken together, these results suggest that ΔFosB-PSMC5 form complexes in NAc which also include CBP/p300 and BRG1 (Fig 4E).

### PSMC5 overexpression increases locomotor responses to cocaine

**Fig 5:**
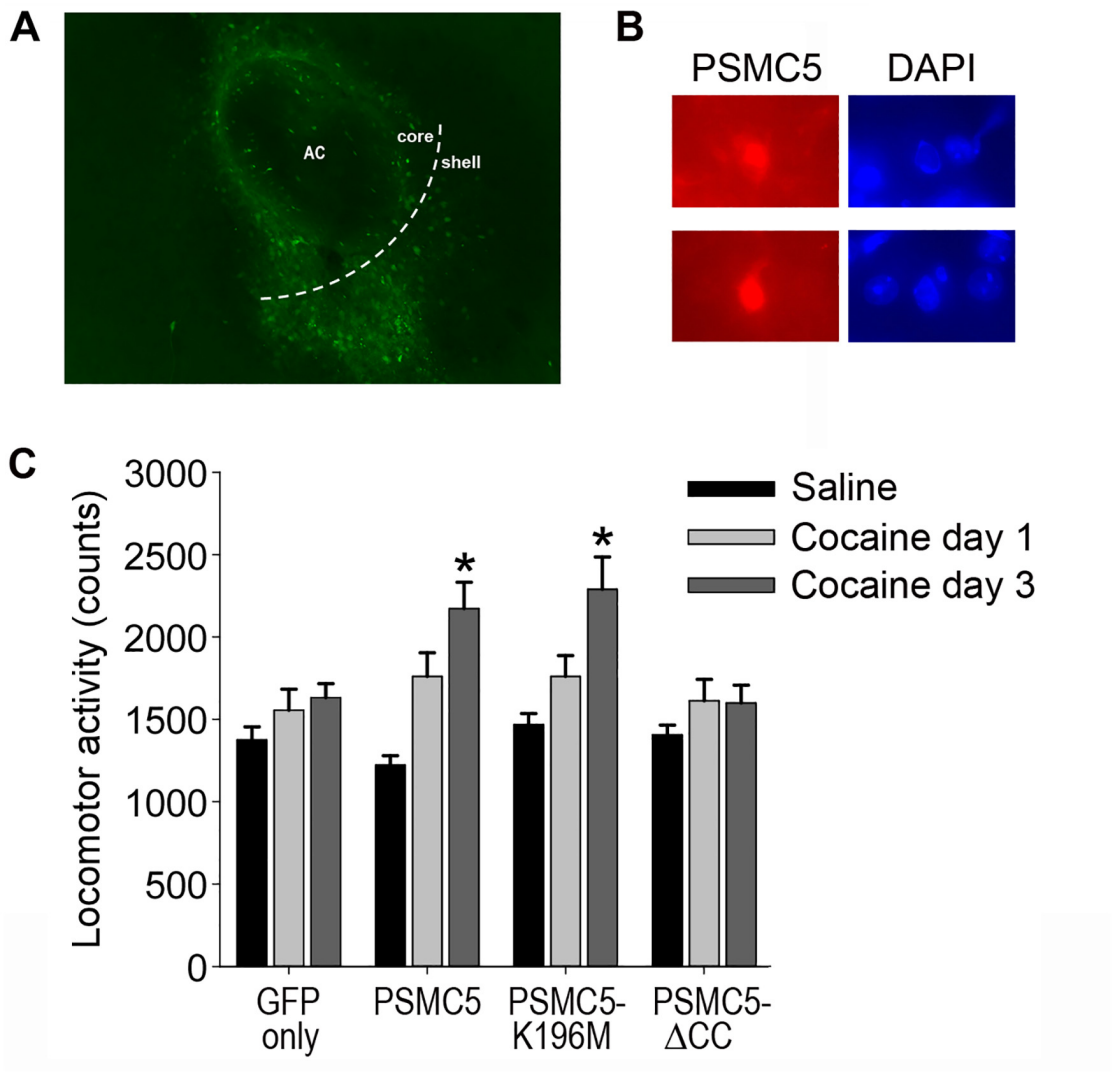
PSMC5 overexpression in NAc increases locomotor responses to cocaine. A. Representative HSV-mediated transgene expression in medial NAc. AC, anterior commissure. NAc core and shell subregions are noted on the figure. B. Representative higher magnifications (60x) of immunohistochemical staining of PSMC5 in NAc neurons after HSV-PSMC5 injection showing that the protein is predominantly nuclear as marked by DAPI staining. C. Mice received bilateral HSV injections into NAc followed by daily IP injections of subthreshold doses of cocaine (7.5 mg/kg). Locomotor responses are shown in response to the first and last of 3 daily doses of the drug. Overexpression of PSMC5 or PSMC5-K196M increases locomotor responses to repeated cocaine, an effect not seen with PSMC5-ΔCC. There was no significant effect of the transgenes on locomotor responses to initial cocaine doses. ANOVA F(3,125) = 4.163, *p<0.05 by Dunnett’s posthoc test.

The prominent binding of PSMC5 with ΔFosB in NAc prompted us to investigate whether increasing PSMC5 levels in this brain region regulates behavioral responses to cocaine. We generated a Herpes Simplex Virus (HSV) vector that overexpresses either wildtype PSMC5 or one of its mutants and validated the vectors in NAc in vivo (Fig 5A). Viral-mediated expression of PSMC5 predominates in the cell nucleus (Fig 5B). Mice overexpressing wild-type PSMC5 did not show altered responses to initial doses of cocaine, but displayed increased locomotor activation in response to repeated cocaine doses compared to GFP-expressing control mice. In contrast, mice overexpressing a mutant form of PSMC5 that lacks its coiled-coil domain (PSMC5-ΔCC) did not exhibit this effect (Fig 5B). Interestingly, overexpression of PSMC5-K196M, which lacks the ATPase activity of the wildtype protein, also potentiated cocaine’s locomotor responses.

## Discussion

The results of the present study reveal a new mechanism by which ΔFosB mediates its effects on the brain and a new mechanism involved in cocaine action. By use of an unbiased approach, a yeast two-hybrid assay, we identified PSMC5 as a novel binding partner for ΔFosB. We validated this finding both in cultured cells in vitro and in the NAc in vivo by demonstrating robust PSMC5-ΔFosB binding. Importantly, nuclear levels of PSMC5 are induced in NAc by chronic cocaine administration. We showed further that PSMC5-ΔFosB binding occurs in concert with several other transcriptional activator proteins, namely CBP and p300 (two HATs) and BRG1 (a key constituent of SWI-SNF chromatin remodeling complexes). Together, our findings support the hypothesis that PSMC5 is part of the transcriptional activation complex that is recruited to at least certain ΔFosB-induced genes during a course of chronic cocaine administration (Fig 4E). Consistent with this hypothesis is the additional finding that overexpression of PSMC5 in NAc, like the overexpression of ΔFosB itself, promotes behavioral responses to cocaine. It would be interesting in future studies to follow up these in vivo observations with characterization of PSMC5-ΔFosB-HAT-BRG1 interactions by use of in vitro reporter assays.

The involvement of PSMC5 in cocaine action is completely novel. Identified initially as a member of a large family of ATPases that comprise the proteasome, PSMC5 has been shown over the years to interact with several transcription factors, including c-Fos, p53, nuclear hormone receptors, and constituents of the basal transcription complex [25], however, few functional studies have been performed over the years [26]. Its best established action is to promote the activity of MYC transcription factors in cultured cells [27]. The implication of PSMC5 in transcriptional mechanisms has suggested a potential role for ubiquitination-proteasomal activity in the regulation of gene transcription, but the involvement of PSMC5 in such regulation remains virtually untested to date [28,29].

Very little is known about PSMC5 function in brain. An earlier study demonstrated widespread expression of PSMC5 mRNA throughout brain [30], but its functional activity has remained unstudied. Our findings now prompt further investigations of this interesting protein to better understand its role in regulating gene transcription and its relationship to ubiquitination-proteasomal function in brain. The binding of PSMC5 to ΔFosB is mediated by PSMC5’s coiled-coil domain. Moreover, the ability of PSMC5 to promote locomotor responses to cocaine, while requiring the coiled-coil domain, does not require the ATPase activity that is intrinsic to the protein. These results raise the possibility that, at least in our system, the main activity of PSMC5 might be mediated through its binding to ΔFosB and other transcriptional regulatory proteins and not through its proteasomal-related activity per se. Further work is needed to directly test this and alternative possibilities. The hypothesis that viral-mediated overexpression of PSMC5 increased locomotor responses to cocaine via interactions with ΔFosB is plausible, despite the use of a 3 day cocaine treatment regimen, because it is known that appreciable levels of ΔFosB accumulate in brain within this time frame [3].

The present findings further substantiate the utility of using unbiased, open-ended experimental approaches in studying the molecular basis of brain regulation. Our initial attention to PSMC5 was based solely on its prominent binding to ΔFosB in a yeast two-hybrid assay, yet it appears to be an important component of transcriptional changes that are recruited in NAc by repeated cocaine administration. A better understanding of the detailed mechanisms by which nuclear levels of PSMC5 are induced by cocaine and, in turn, by which PSMC5 then contributes to cocaine-induced transcriptional activation complexes are the focus of current investigations. Meanwhile, our yeast two-hybrid assay revealed several additional putative binding partners of ΔFosB (Table 1) which also now warrant direct examination in cocaine models. Together, this work is contributing to an increasing understanding of the complex molecular mechanisms through which cocaine alters NAc function.
