# Correction: PSMC5, a 19S Proteasomal ATPase, Regulates Cocaine Action in the Nucleus Accumbens

**DOI:** 10.1371/journal.pone.0131263

**Published:** 2015-06-25

**Authors:** Yoko H. Ohnishi, Yoshinori N. Ohnishi, Takanori Nakamura, Mizuki Ohno, Pamela J. Kennedy, Yasuyuki Ohkawa, Akinori Nishi, Rachael Neve, Teruhisa Tsuzuki, Eric J. Nestler

The sixth author's first and last name are incorrectly switched. The correct name is Yasuyuki Ohkawa. The correct citation is: Ohnishi YH, Ohnishi YN, Nakamura T, Ohno M, Kennedy PJ, Ohkawa Y, et al. (2015) PSMC5, a 19S Proteasomal ATPase, Regulates Cocaine Action in the Nucleus Accumbens. PLoS ONE 10(5): e0126710. doi:10.1371/journal.pone.0126710
